# Clinical Profiles and Survival Outcomes of Patients With Relapsed Ovarian Cancer: A Single-Center Study

**DOI:** 10.7759/cureus.74724

**Published:** 2024-11-29

**Authors:** Jiss Joy, Kannan J, Satheesh Kumar, Mohamed Arshad

**Affiliations:** 1 Medical Oncology, Madras Medical College, Chennai, IND

**Keywords:** high-grade serous ovarian carcinoma, optimal cytoreduction, platinum-resistant relapse, platinum-sensitive relapse, recurrent ovarian cancer, suboptimal cytoreduction

## Abstract

Background

Ovarian cancer is the third most prevalent form of cancer among women in India. The majority of patients are diagnosed at an advanced stage. Many women with late-stage ovarian cancer experience a recurrence and need subsequent treatment, even after initial therapy. The prognosis of patients who experience relapse after treatment is poor. The objective of this study was to assess the clinical characteristics and survival rates of individuals diagnosed with relapsed epithelial ovarian cancer (EOC).

Methods

This retrospective study included patients with a relapse of epithelial ovarian carcinoma who were treated at the Department of Medical Oncology, Institute of Obstetrics and Gynecology (IOG), Madras Medical College, Chennai, India, over a period of six years, from January 2015 to December 2020.

Results

Sixty-six patients were enrolled in this study. The median patient age was 52 years. Most patients were postmenopausal (66.67%, n=44) and multiparous (75.76%, n=50). The most common histological type was serous (72.72%, n=48), and the majority of patients were in the later stages of the illness (83.34%, n=55). The median post-relapse survival of the study population was 23 months (95% confidence interval (CI): 20.61-25.39). The median survival of patients who were upfront stage I, II, III, and IV was 30, 13, 23, and nine months, respectively (p<0.05). Patients who underwent optimal cytoreduction had better survival rates than those who did not (28 versus 18 months, p<0.05). The median post-relapse survival was better in the platinum-sensitive group than in the platinum-resistant group (26 versus 16 months, p<0.05). Patients with a single relapse site had better survival rates than those with multiple sites of recurrence (26 versus 13 months, p<0.05). Patients with mucinous histology showed maximum survival (p<0.05). Individuals who initially underwent surgery had superior median survival rates following relapse compared to those who received neoadjuvant chemotherapy as their first treatment (25 versus 23 months, p=0.404). Cox regression analysis revealed that platinum-sensitive patients were 4.204 times more likely to survive than platinum-resistant patients. Similarly, those presenting with single lesions were 7.275 times more likely to survive for a longer time than those with multiple lesions.

Conclusion

This study emphasizes the importance of achieving optimal cytoreduction and underscores the prognostic significance of platinum sensitivity and recurrence patterns in patients with relapsed ovarian cancer. Late detection can result in unfavorable outcomes.

## Introduction

Among women globally, ovarian cancer ranks as the eighth most prevalent malignancy and is the eighth leading cause of cancer-related mortality [1]. In India, it is third in terms of incidence and mortality in women [2]. Despite improvements in surgical techniques and systemic therapies, many patients continue to experience relapses. Most patients (70%-80%) are diagnosed at an advanced stage, resulting in poor long-term survival rates (15%-30%) [3]. On the other hand, patients diagnosed at an early stage of the disease have a much more favorable outlook, with over 80% surviving the disease [4]. The main treatment modalities are surgery and systemic therapy. Surgery is the definitive treatment for ovarian carcinomas. It includes a primary cytoreduction procedure performed upfront and an interval debulking surgery performed after neoadjuvant chemotherapy. Surgery is done to stage the disease and achieve optimal cytoreduction [5]. Neoadjuvant chemotherapy is administered in inoperable cases prior to surgery. The order of these treatment approaches is influenced by factors such as the disease status, whether surgery is feasible, and the overall health condition of the patient [6-8].

Significant progress has been achieved in treating ovarian cancer, leading to improved outcomes for patients. Newer modalities, such as vascular endothelial growth factor (VEGF) inhibitors, poly ADP-ribose polymerase (PARP) inhibitors, intraperitoneal chemotherapy, hyperthermic intraperitoneal chemotherapy (HIPEC), and pressurized intraperitoneal aerosol chemotherapy (PIPAC) have shown promising results [9-13]. However, due to resource constraints, many of these modalities are not available in developing countries, such as India.

By focusing on relapsed ovarian cancer, this research seeks not only to enhance clinical outcomes but also to contribute to a growing body of knowledge that addresses the unique needs of patients in resource-limited settings. The findings of this study could pave the way for more effective management strategies and improved support mechanisms, ultimately leading to a better quality of life for women with this formidable disease. The objective of this research was to investigate the clinical characteristics and therapeutic results in patients experiencing a recurrence of epithelial ovarian cancer (EOC).

## Materials and methods

This was a retrospective study of patients with EOC who relapsed after achieving complete remission after treatment with surgery and chemotherapy at the Institute of Obstetrics and Gynecology (IOG), Madras Medical College, Chennai, India. Patients with primary peritoneal carcinomatosis and fallopian tube cancer were also included. The study included individuals who received treatment during a six-year timeframe, spanning from January 2015 to December 2020. Data were retrieved from a database maintained by the Department of Medical Oncology. Information on patient characteristics, medical conditions, initial staging, therapeutic interventions, and subsequent monitoring was collected and analyzed.

The study examined data from individuals meeting specific criteria. These included a verified diagnosis of epithelial ovarian cancer (encompassing stages I-IV as per the International Federation of Gynecology and Obstetrics (FIGO) classification), as well as primary peritoneal and fallopian tube cancers. The subjects had experienced a recurrence of the disease after initially achieving a complete response following surgery and treatment with platinum-based combination chemotherapy. The study excluded individuals with incomplete records and those diagnosed with non-epithelial ovarian malignancies.

Complete eradication of all clinical and radiological pathological abnormalities was considered a complete response. CA-125 serum tumor marker level of 35 U/mL or below was taken as normal [14]. Recurrence was defined as the emergence of a clinical or radiological lesion, which was subsequently confirmed through histopathological examination and correlated with CA-125 levels. The FIGO international staging system was utilized for staging purposes [15]. The duration between relapse and death from any cause was used to define post-relapse survival. Optimal cytoreduction was defined as a residual tumor of 1 cm or less [16]. Patients with a platinum-free interval of six months or longer are considered to have platinum-sensitive disease.

Data analysis

Initially, the information was entered into Microsoft Excel (Microsoft Corp., Redmond, WA). Data analysis was performed using version 25.0 of IBM SPSS Statistics for Windows (IBM Corp., Armonk, NY). Frequencies and percentages were employed to represent categorical variables. Quantitative variables, such as age, were represented in terms of median and interquartile range, and mean and standard deviation. The Kaplan-Meier method was employed to assess survival, while the log-rank test was utilized for comparing groups. The duration from recurrence to death from any cause was used to measure post-relapse survival. At the final follow-up, patients who had not experienced any events were censored. To examine the impact of covariates on survival, a multivariate Cox regression analysis was conducted. Findings were deemed statistically significant when the p-value fell below 0.05.

## Results

This study used the medical records of 66 individuals who experienced a significant recurrence of epithelial ovarian cancer. The clinical profiles of the patients are shown in Table 1.

**Table 1:** Clinical profiles of patients

Characteristics	
Age (years)	
Mean	51.58
Median	52
Range	23-72
Menopausal status (number (%))	
Premenopausal	22 (33.33)
Postmenopausal	44 (66.67)
Parity (number (%))	
Nulliparous	9 (13.64)
Uniparous	7 (10.60)
Multiparous	50 (75.76)
Histology (number (%))	
Serous	48 (72.72)
Endometrioid	7 (10.60)
Mucinous	5 (7.58)
Clear cell	4 (6.06)
Others	2 (3.03)
Stage at diagnosis (number (%))	
I	7 (10.60)
II	4 (6.06)
III	52 (78.79)
IV	3 (4.55)

Our patient group had a median age of 52 years, with ages spanning from 23 to 72 years. Following relapse, the median follow-up duration was 24 months. The majority of patients were women who had undergone menopause and were multiparous. The most common histological type identified was serous carcinoma, with a significant proportion of patients presenting with advanced stages of the disease. The patients’ treatment and relapse details are presented in Table 2.

**Table 2:** Treatment and relapse details of patients

Treatment history	Number (%)
Primary treatment	
Upfront surgery	22 (33.33)
Interval debulking surgery	44 (66.67)
Extent of surgery	
Optimal cytoreduction	38 (57.58)
Suboptimal cytoreduction	28 (42.42)
Type of relapse	
Platinum-sensitive	44 (66.67)
Platinum-resistant	22 (33.33)
Number of recurrence sites	
Single	45 (68.18)
Multiple	21 (31.81)

The primary treatment approach for most patients (66.67%, n=44) involved initial chemotherapy, three or four cycles, followed by interval debulking surgery. Optimal cytoreduction was achieved in 57.58% (n=38) of patients. Patients in the neoadjuvant chemotherapy group completed their chemotherapy cycles after the interval debulking surgery. Patients who underwent upfront surgery were administered adjuvant chemotherapy. All patients were administered injection paclitaxel 175 mg/m^2^ and injection carboplatin area under the curve (AUC) 5 in the neoadjuvant and adjuvant setting. At relapse, 66.67% (n=44) of patients had platinum-sensitive disease. These patients were re-challenged with platinum-based chemotherapy (paclitaxel/carboplatin, gemcitabine/carboplatin, or pegylated liposomal doxorubicin/carboplatin) [17]. Platinum-resistant patients were treated with single-agent chemotherapy, including weekly paclitaxel, pegylated liposomal doxorubicin, gemcitabine, and topotecan [18]. Further lines of chemotherapy were administered based on drugs that were not previously administered upon progression and the performance status of the patients.

**Figure 1:**
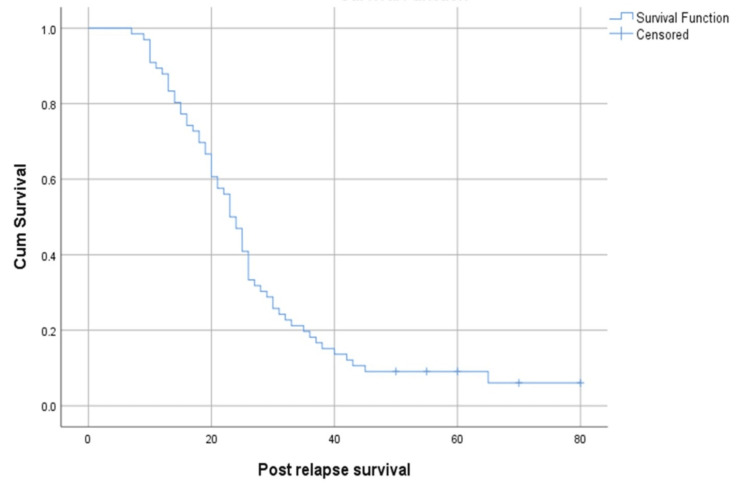
Kaplan-Maier curve depicting post-relapse survival

At the time of evaluation, 61 of the 66 patients had passed away, with only five remaining alive. The median post-relapse survival time of the study population was 23 months (95% confidence interval (CI): 20.61-25.39) (Figure 1).

**Figure 2:**
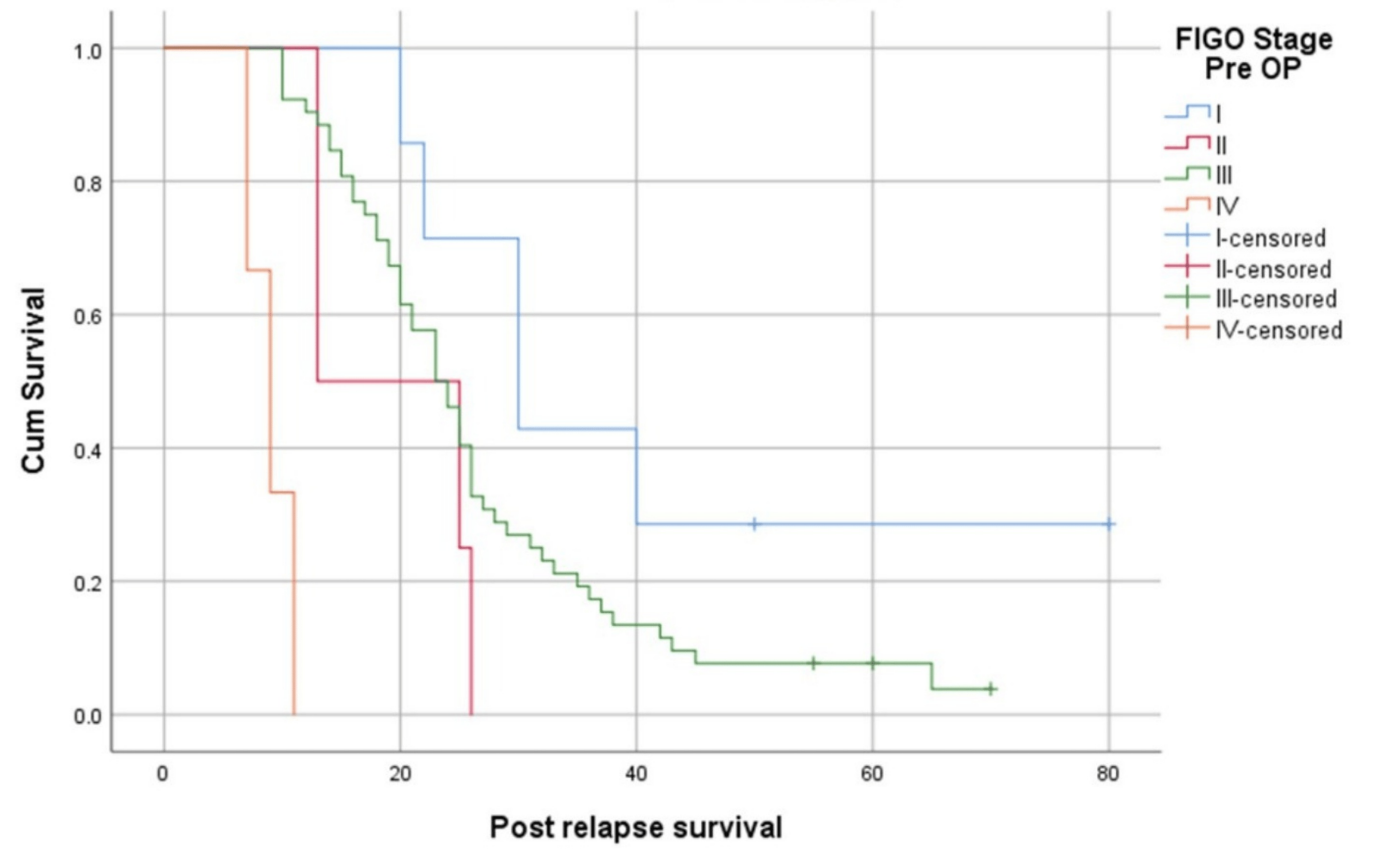
Survival function based on the stage of the disease at presentation FIGO: International Federation of Gynecology and Obstetrics

The median post-relapse survival of patients who were upfront stage I, II, III, and IV was 30, 13, 23, and nine months, respectively (p<0.05) (Figure 2). However, it should be noted that the number of patients in the earlier stages of the disease was low.

**Figure 3:**
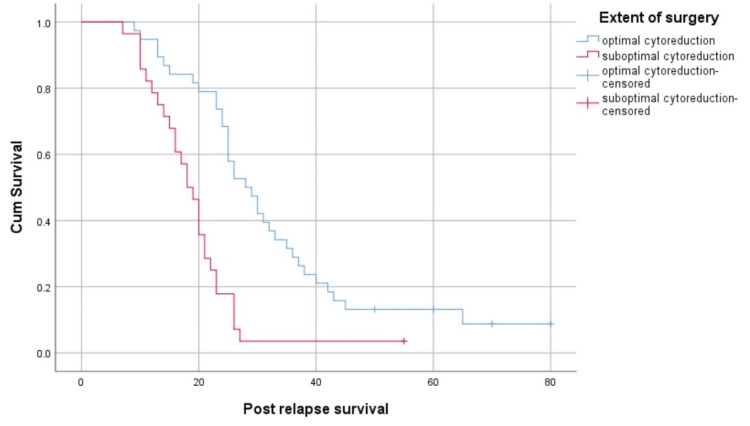
Survival function based on the completeness of surgery

Patients who underwent optimal cytoreduction had a better median post-relapse survival than those who underwent suboptimal surgery (28 months (95% CI: 22.97-33.03) versus 18 months (95% CI: 15.41-20.59), p<0.05) (Figure 3).

**Figure 4:**
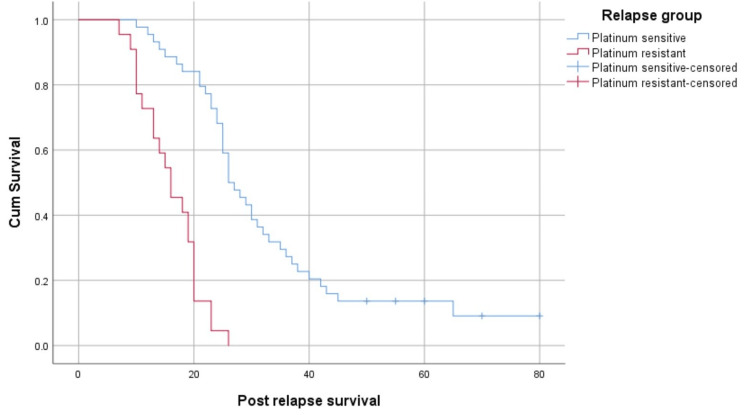
Survival function based on the type of relapse

The median post-relapse survival was better in the platinum-sensitive group than in the platinum-resistant group (26 months (95% CI: 22.29-29.72) versus 16 months (95% CI: 11.42-20.58), p<0.05) (Figure 4).

**Figure 5:**
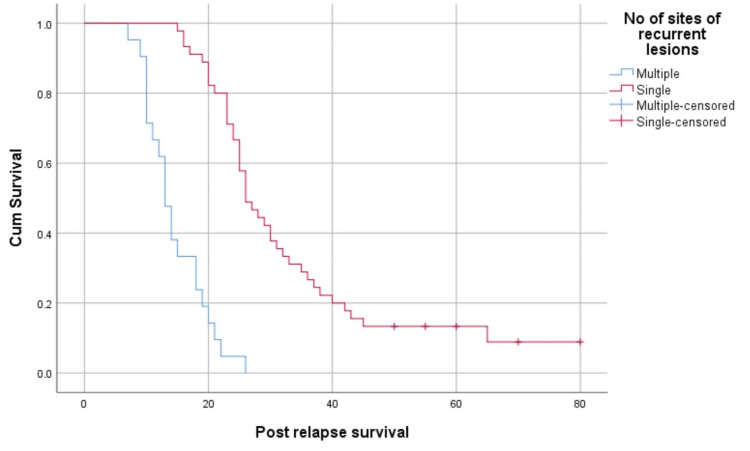
Survival function based on the number of recurrent lesions

Patients with a single recurrence site had better median post-relapse survival than those with multiple sites of recurrence (26 months (95% CI: 22.71-29.29) versus 13 months (95% CI: 11.21-14.79), p<0.05) (Figure 5).

**Figure 6:**
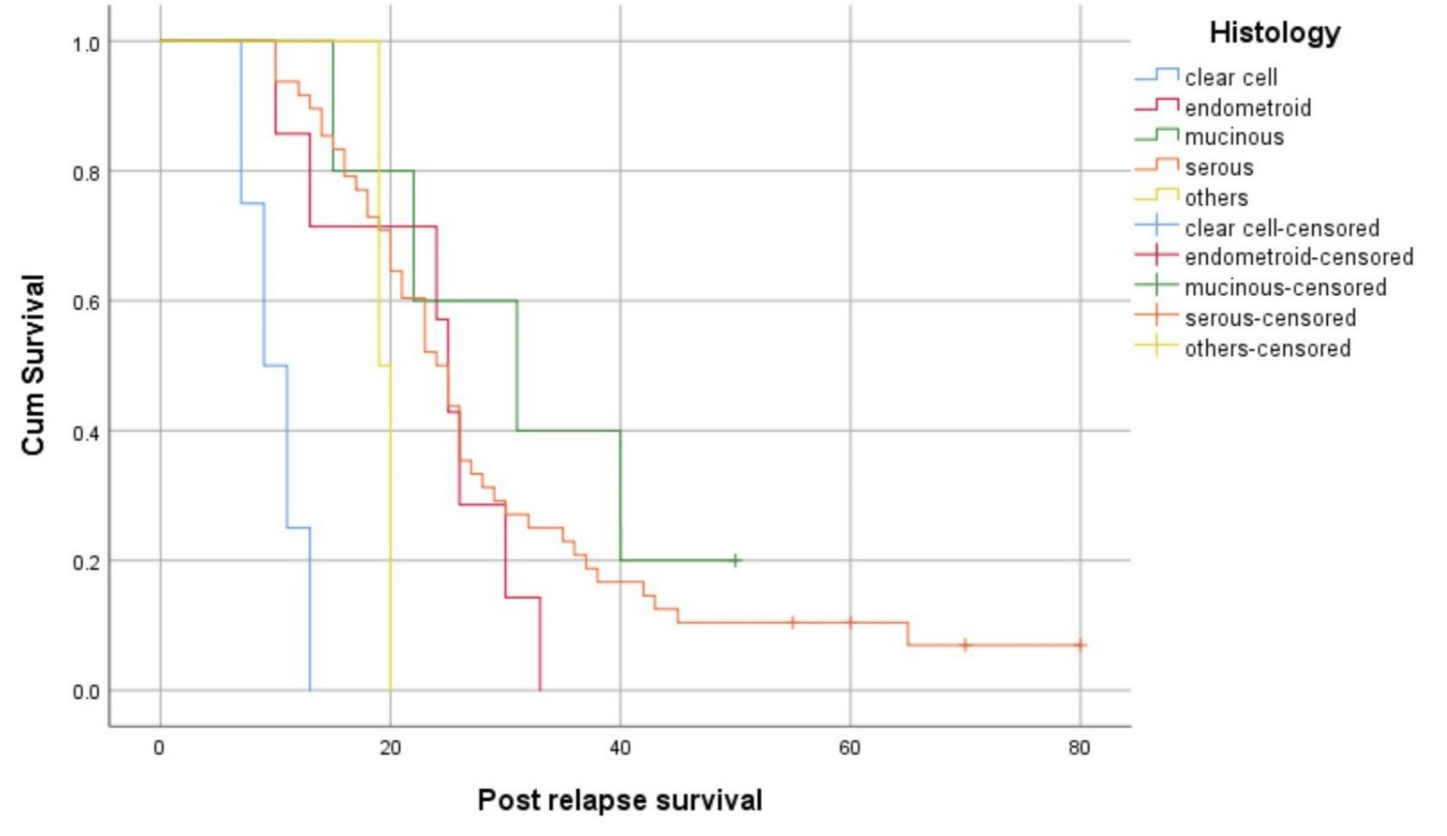
Survival function based on histological subtype

Mucinous histology had the maximum median post-relapse survival, followed by endometroid, serous, others, and clear cell histology (31, 25, 24, 19, and nine months, respectively; p<0.05) (Figure 6).

**Figure 7:**
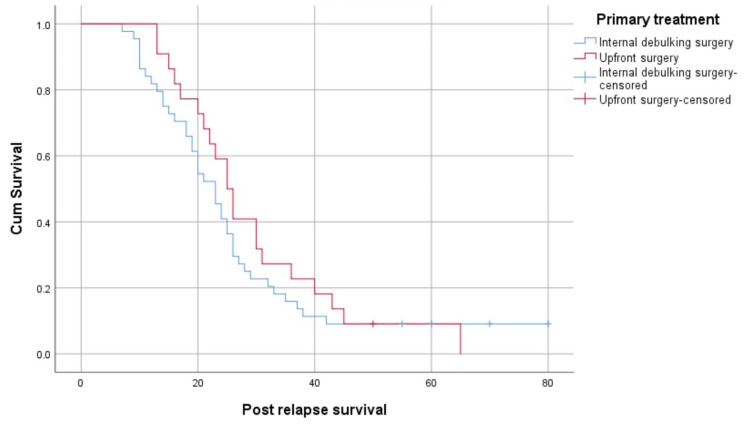
Survival fraction based on the timing of surgery

Patients who underwent upfront surgery had a better median post-relapse survival than those who underwent neoadjuvant chemotherapy, but this difference was not statistically significant (25 months (95% CI: 22.71-29.29) versus 23 months (95% CI: 11.21-14.79), p=0.404) (Figure 7).

According to the multivariate Cox proportional hazards model (Table 3), patients who were platinum-sensitive had a 4.204 times higher likelihood of survival compared to those who were platinum-resistant. Similarly, those presenting with single lesions were 7.275 times more likely to survive longer than those with multiple lesions. The extent of surgery, primary treatment, histology, and stage had no effect on survival.

**Table 3:** Multivariate Cox regression model showing independent predictors of survival In the Cox regression analysis, the p-value was considered statistically significant if it was ≤0.05. FIGO: International Federation of Gynecology and Obstetrics

Variables	Odds ratio	95% confidence interval		p-value
		Lower	Upper	
Relapse group	4.204	1.919	9.206	<0.050
Extent of surgery	1.567	0.812	3.024	0.180
Number of sites of recurrent lesions	7.275	3.539	14.957	<0.050
Primary treatment	1.171	0.622	2.206	0.624
Histology	1.516	0.807	2.850	0.196
FIGO stage	0.593	0.254	1.381	0.225

## Discussion

In both national and international cancer registries, ovarian cancer ranks among the top 10 most common malignant diseases. Epithelial cells are the source of the vast majority (95%) of ovarian cancers [19]. The remaining 5% originate from other cell types within the ovary, specifically germ cell and sex cord-stromal tumors. The majority of individuals diagnosed with epithelial ovarian cancer are found to be in an advanced stage of the disease. In this retrospective study, we analyzed the clinical profiles and survival outcomes of patients who relapsed after primary treatment.

The median age group of our patients was 52 years, which is similar to what was observed in other Indian studies [20,21]. This was earlier than the median age in Western countries, which is between 60 and 64 years [22]. Many women with advanced-stage ovarian cancer relapse despite undergoing surgery and platinum-based doublet chemotherapy. The chance of relapse depends mainly on the upfront stage, completeness of surgery, and adjuvant treatment. Those who relapse require the next line of treatment, including chemotherapy and secondary cytoreduction, in selected cases.

In this study, 83.3% of the patients who relapsed after primary treatment were in the advanced stages of the disease (stages III and IV), which again substantiates that patients in the advanced stage of the disease are more prone to relapse. Most of the relapsed cases had a serous histology, possibly because it is the most common pathology. We found significant differences in survival with respect to histology, with the mucinous type having the longest median post-relapse survival of 31 months, endometroid of 25 months, serous of 24 months, and the least clear cell type of nine months. There were two cases of carcinosarcoma with a median post-relapse survival of 19 months. However, this should be interpreted with caution, as there were only a few cases in other histological subgroups, apart from the serous subtype. Patients with stage I disease upfront had the maximum survival after relapse. Decreased survival was observed with advancing disease stages.

Platinum sensitivity at relapse was an important factor that correlated with survival in patients in the platinum-sensitive group who survived longer. Most of those who were platinum-sensitive responded well to platinum re-challenge and had a longer survival. The number of relapse sites is another factor that is correlated with survival. Patients who had only a single-site relapse had better survival. Patients with recurrence at a single site demonstrated a higher likelihood of successful disease management through chemotherapy. For individuals with platinum-sensitive recurrent ovarian cancers and a favorable Arbeitsgemeinschaft Gynäkologische Onkologie (AGO) score defined as an Eastern Cooperative Oncology Group performance status score of 0 (on a 5-point scale), ascites of less than 500 mL, and complete resection at initial surgery, undergoing secondary cytoreductive surgery has been associated with improvements in both overall survival and progression-free survival [23]. However, none of the patients in the platinum-sensitive group in this study were treated with secondary cytoreductive surgery, which is a major drawback observed in this study. Survival could have been better if the patients had undergone secondary cytoreduction at platinum-sensitive relapse. The completeness of surgery is another important factor for survival [24]. Patients who had optimal cytoreduction had better survival than those who had significant residual disease. There was no difference in whether the surgery was performed upfront or after neoadjuvant chemotherapy. The main factor contributing to survival was the completeness of the surgery. The median post-relapse survival in our population was 23 months, which is lower than that of patients in Western countries [25]. This may be due to the advanced stage of the disease in which patients presented to our center and the unavailability of the latest treatment modalities.

The testing facility for BRCA mutations and homologous recombination deficiency (HRD) testing was not available at our institute and was not covered under any schemes at that time. Therefore, the procedure was not performed. Numerous studies on advanced ovarian cancer have demonstrated improved survival outcomes with the use of VEGF inhibitors (bevacizumab) and PARP inhibitors [11,13]. However, the patients did not have access to these drugs owing to resource constraints, which could have led to inferior results compared with the Western population.

This study has some limitations. The records of many patients were incomplete, and follow-up details were missing. Therefore, many patients were excluded from this study. The number of patients in the earlier stages of the disease and certain histologies was very low. Therefore, their correlation with survival should be cautiously interpreted. None of the patients underwent a secondary cytoreduction. The patients did not have access to newer drugs.

## Conclusions

To summarize, ovarian cancer continues to be one of the deadliest cancers affecting women, especially in developing countries with limited resources, such as India. Despite the introduction of new treatment approaches, chemotherapy remains the primary systemic therapy available to most patients in these regions. This study highlights the critical role of achieving optimal cytoreduction and underlines the prognostic importance of platinum sensitivity and recurrence patterns in patients with recurrent ovarian cancer. A delayed diagnosis can lead to poor outcomes.
